# Colonic Leucine-Rich Repeat Kinase 2 Expression Is Increased and Associated With Disease Severity in Patients With Parkinson’s Disease

**DOI:** 10.3389/fnagi.2021.819373

**Published:** 2022-01-17

**Authors:** Peng-Hsiang Liao, Han-Lin Chiang, Chia-Tung Shun, Jen-Fan Hang, Han-Mo Chiu, Ming-Shiang Wu, Chin-Hsien Lin

**Affiliations:** ^1^College of Medicine, National Taiwan University, Taipei, Taiwan; ^2^College of Medicine, Graduate Institute of Clinical Medicine, National Taiwan University, Taipei, Taiwan; ^3^Department of Neurology, Neurological Institute, Taipei Veterans General Hospital, Taipei, Taiwan; ^4^Department of Pathology, National Taiwan University Hospital, Taipei, Taiwan; ^5^Department of Pathology and Laboratory Medicine, Taipei Veterans General Hospital, Taipei, Taiwan; ^6^Department of Internal Medicine, National Taiwan University Hospital, Taipei, Taiwan; ^7^Department of Integrated Diagnostics and Therapeutics, National Taiwan University Hospital, College of Medicine, National Taiwan University, Taipei, Taiwan; ^8^Department of Neurology, National Taiwan University Hospital, College of Medicine, National Taiwan University, Taipei, Taiwan

**Keywords:** Parkinson’s disease, colon biopsy, leucine-rich repeat kinase 2, inflammatory bowel diseases, biomarker (BM)

## Abstract

**Background:**

Mutations in leucine-rich repeat kinase 2 (*LRRK2*) comprise a common genetic risk factor for Parkinson’s disease (PD) and inflammatory bowel disease (IBD). We investigated the expression of LRRK2 in colonic biopsies obtained from a cohort of PD patients and healthy controls.

**Methods:**

A cohort of 51 PD patients and 40 age- and gender-matched controls who have colonic biopsied samples were recruited. Among these participants, 26 individuals (12 PD patients and 14 controls) had a series of colon biopsies. For the patients with PD, the first biopsies were taken before the PD diagnosis. The colonic expression of LRRK2 was assayed by immunohistochemical staining.

**Results:**

The fraction of LRRK2-positive cells among the total cell count in biopsied colonic tissues was significantly higher in PD patients than controls (0.81% ± 0.53% vs. 0.45% ± 0.39%; *P* = 0.02). Colonic LRRK2 immunoreactivity was higher in those with *LRRK2* genetic variants compared to those with wild type *LRRK2* (2.44% ± 1.15% vs. 0.21 ± 0.13%, *P* < 0.01). Age had no effect on LRRK2 expression (*P* = 0.96). LRRK2 expression correlated with disease severity in regards to motor symptoms measured by the UPDRS part III scores (r = 6335, *P* < 0.001) and cognitive dysfunction measured by the mini-mental status examination scores (*r* = -0.5774, *P* < 0.001). PD patients in the prodromal phase had a steeper increase in colonic LRRK2 expression compared to controls during the serial colon biopsy assessment (*P* < 0.01).

**Conclusion:**

Colonic LRRK2 expression was increased in PD patients compared to controls, and the expression level correlated with disease severity.

## Introduction

The pathological hallmarks of Parkinson’s disease (PD) are progressive dopaminergic neuron loss and the presence of intracellular α-synuclein aggregations, termed Lewy bodies (Kon et al., 2020). Post-mortem studies have shown that the misfolded α-synuclein in PD may start in the enteric nervous system (ENS) of the gastrointestinal tract and subsequently propagate into brain stem nuclei via cell-to-cell transmission through the vagus nerve (Braak et al., 2003a,b). These pathological findings are coherent with clinical observations that the most common preceding symptom of PD is gastrointestinal dysfunction, particularly constipation, which may appear decades prior to the onset of motor symptoms (Savica et al., 2009). These observations prompted the hypothesis that PD is caused by an environmental trigger that alters the gut microenvironment to initiate a pathological process leading to central dopaminergic neurodegeneration through the gut-brain axis (Houser and Tansey, 2017).

Based on this gut-brain axis theory, α-synuclein immunostaining in the gut has received attention as a potential biomarker. However, many of these reports have raised concerns regarding the specificity of enteric α-synuclein expression because its immuno-reactivity has also been observed in healthy individuals and the results are conflicting (Derkinderen et al., 2011; Olanow et al., 2014). These findings suggest that enteric mucosal α-synuclein may play a limited role as a diagnostic biomarker for PD (Visanji et al., 2015; Antunes et al., 2016; Chung et al., 2016). Additionally, the investigation of enteric α-synuclein expression in a cross-sectional design has not contributed further insights into the specific propagation or accumulation of α-synuclein in the disease process of PD. Therefore, a need exists for alternative gastrointestinal biomarkers that reflect disease progression in PD.

Recent epidemiological and genetic evidence has linked PD to inflammatory bowel disease (IBD). Mutations in *Leucine-Rich Repeat Kinase* 2 (*LRRK2*) are the most common genetic factors in familial PD patients (Zimprich et al., 2004). Recent genome-wide association studies (GWAS) also highlighted *LRRK2* is an important risk factor for sporadic PD patients (Chang et al., 2017; Foo et al., 2017). Notably, GWAS have shown that *LRRK2* is also a susceptibility gene for IBD, and IBD is associated with an increased risk of PD (Umeno et al., 2011; Zhu et al., 2019). This genetic link between PD and IBD prompted us to investigate a potential association between the expression levels of LRRK2 in colonic biopsies from patients with PD. Here, we investigated the expression of LRRK2 in routine colonic biopsies from a longitudinal follow-up cohort of PD patients and healthy controls to investigate the changes in LRRK2 expression with the disease course in PD.

## Materials and Methods

### Study Participants

A total of 51 patients with PD and 40 healthy controls who have colon biopsied samples were recruited from National Taiwan University Hospital. Patients were diagnosed with PD according to the United Kingdom PD Society Brain Bank criteria. All patients had received a regular health check-up with colonoscopy. Control participants who required endoscopic colonoscopies for routine medical screening or abdominal discomfort were recruited from the outpatient clinic of the Department of Gastroenterology of the same hospital. Participants were excluded if they were diagnosed with colon cancer, IBD, irritable bowel syndrome, or use of non-steroidal anti-inflammatory drugs, antibiotics, or probiotics within 3 months of specimen collection. All patients with PD were evaluated using the Unified Parkinson’s disease Rating Scale part III motor scores (UPDRS-III) and Hoehn-Yahr staging. Neuropsychological evaluations were carried out with the mini-mental state examination (MMSE). All control participants underwent a detailed neurological examination to rule out PD symptoms and cognitive impairment.

Among the 91 participants, 26 (12 PD patients and 14 control participants) underwent a series of colonoscopies with colon biopsies to follow-up on the development of colon polyps. For the patients with PD, the first biopsies were taken before the PD diagnosis. For these 26 participants with serial colonic biopsies, the latest biopsied samples were used for the group comparison between PD patients and controls. We categorized the participants who underwent serial colonic biopsies into five groups: serial group 1, colonic biopsies ≥ 8 years before the onset of motor symptoms (≤-8 years); serial group 2, colonic biopsies 3–7 years before the onset of motor symptoms (-7 to -3 years); serial group 3, colonic biopsies 2 years before or after the onset of motor symptoms (-2 to + 2 years); serial group 4, colonic biopsies 3–7 years after the diagnosis of PD (+ 3 to + 7 years); and serial group 5, colonic biopsies ≥ 8 years after the onset of motor symptoms (≥+8). The control participants were classified into five groups according to the interval between the biopsied age and the mean age at which PD was diagnosed in the enrolled PD patients. The study protocol was approved by the Institutional Board Committee of National Taiwan University Hospital. Written informed consent was obtained from each participant.

### Immunohistochemistry of Biopsied Colonic Tissues

We retrospectively examined the colonic biopsy specimens from patients with PD and controls after they provided written informed consent to participate in the study. Three to four random biopsies were taken from each study participant during colonoscopy using standard biopsy forceps. Immediately after sampling, the tissues were fixed in 4% phosphate-buffered formaldehyde and processed for paraffin embedding. The paraffinized tissue blocks were cut by a microtome into 6-μm-thick sections for immunohistochemical staining. Immunohistochemistry was performed with the BioGenex detection system according to the manufacturer’s protocol. Briefly, the paraffin was melted by sequentially incubating the sections in xylene for 30, 5, and 5 min, followed by 5 min each in 100, 95, and 70% alcohol for rehydration. Heat-mediated antigen retrieval was performed in citrate buffer (pH 6.0). Samples were allowed to cool at room temperature for at least 30 min. Protein blocking was performed for 1 h to prevent non-specific antibody binding. A primary antibody against LRRK2 (Abcam, ab1334741, 1:1000) was diluted to the desired concentration, and then added to the mixture and incubated at 4°C overnight. The next day, samples were incubated in hydrogen peroxide and methanol for 15 min to block endogenous peroxidase activity. The samples were then incubated in super enhancer solution and horseradish peroxidase for 30 min each. The 3,3′-diaminobenzidine (DAB) chromogen was added for color development. Nuclei were stained with hematoxylin. Samples were then dehydrated in different grades of alcohol (70, 95, and 100%), followed by incubation in xylene (twice, 3 min each) and a xylene substitute (NeoClear^®^, Merck). Sections were air dried and then mounted on slides.

### Image Analysis and Quantification of Leucine-Rich Repeat Kinase 2 Expression

**FIGURE 1:**
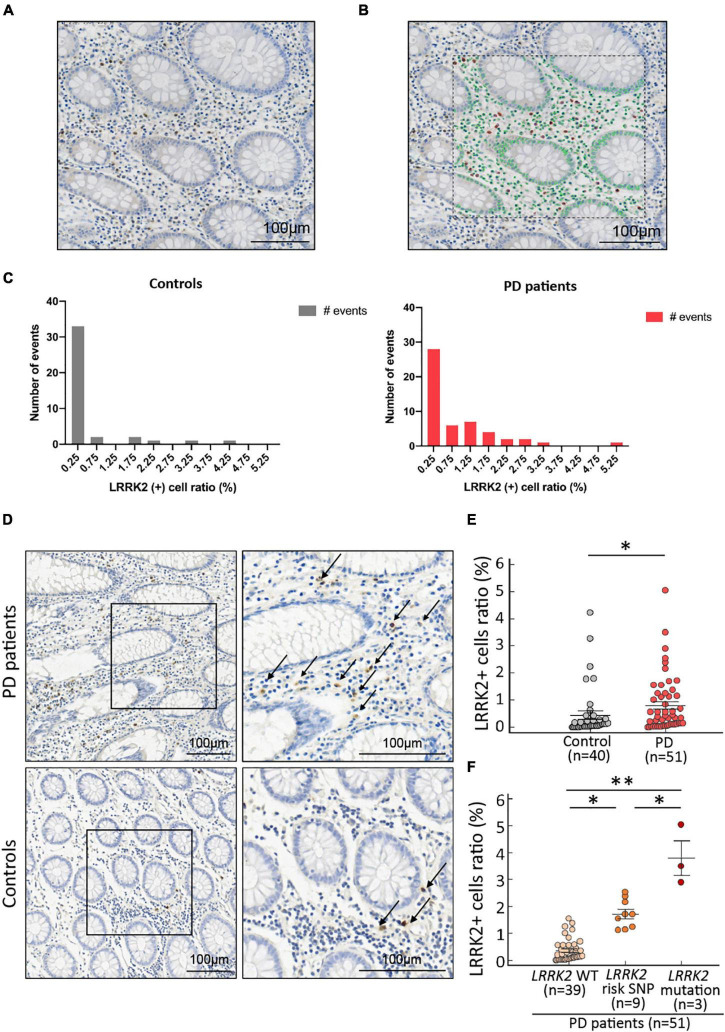
LRRK2 expression in colonic biopsies from PD patients and age- and gender-matched controls. **(A)** Original view of the represented colonic biopsy tissues. **(B)** All nuclei are circled with green lines, and the LRRK2-positive staining detected as positive events are circled in red lines (arrows) by the StrataQuest Analysis software version 6 (TissueGnostics, Vienna, Austria). **(C)** Distribution histograms of the LRRK2-positive ratio in PD patients and controls. **(D)** Representative pictures of LRRK2-positive cells in the submucosa of colonic samples from one 72-year-old male PD patient and one 71-year-old male control participant. Positive events are indicated by arrows. The pictures on the right are the magnification of the framed areas on the left. Scale bar: 100 μm. **(E)** The fractions of LRRK2-positive cells in the total cell counts in colonic biopsies were significantly higher in PD patients than controls (*P* = 0.004 by Mann-Whitney *U*-test). **(F)** The fractions of LRRK2-positive cells in the total cell counts in colonic biopsies from PD patients with wild type or genetic variant in *LRRK2*. *P < 0.05; **P < 0.01.

Slides were scanned using the Aperio Scanscope XT high-resolution scanner at 40 × magnification (Leica Biosystems, Mt. Waverley, Australia). The scanned files were imported to a computer and analyzed using StrataQuest Analysis software version 6 (TissueGnostics, Vienna, Austria). An experience pathologist (CT Shun) who was blinded to the clinical diagnosis examined each biopsied colonic tissue for staining quality and selected region of interests (ROIs) to avoid areas with colon polyp hyperplasia changes. Each slide was then carefully evaluated by two raters (PH Liao and HL Chiang) to choose ROIs comprising areas without abnormal colon polyp tissues for StrataQuest analysis (Figures 1A,B). The scatter plots were created to visualize the positive cells in the source images automatically using the real-time back-gating feature in the software. The cut-off values were designated to discriminate between false events and specific signals based on cell size and staining intensity (Schmid et al., 2015). Within each image, unwanted precipitate staining or areas with poor staining quality were excluded with exclusion masks automatically by the software. Only the mucosa and part of the submucosa areas were included for analysis. Cells that stained positive with both DAB and hematoxylin were counted as positive cells. The total cell count included all cells in unmasked areas within the ROI. The fraction of positive cells among the total cell count was calculated for each ROI.

### Genotyping of Risk Variant and Common Mutations in *Leucine-Rich Repeat Kinase 2*

Genomic DNA was extracted from 10 ml of venous blood using standard protocols. The *LRRK2* c.4322G > A (p. R1441H) mutation on exon 31, *LRRK2* c.6035T > C (p.I2012T) and *LRRK2* c.6055G > A (p.G2019S) mutations on exon 41 and *LRRK2* c.7153G4A (p.G2385R) risk polymorphism on exon 48 were genotyped by direct DNA sequencing as previously described (Lin et al., 2008).

### Statistical Analysis

Continuous variables were expressed as the mean ± standard deviation. Categorical data were expressed as the number and percentage. Continuous variables were compared between groups using the two-sample Student test, or the non-parametric Mann-Whitney U test when normality could not be assumed. Statistical analyses were performed in GraphPad Prism 8.0 (San Diego, California, United States). *P*-values < 0.05 were considered significant.

## Results

Fifty-one patients with PD (mean age at colon biopsy, 69.2 ± 9.2 years; 55% were men) and 40 age- and gender-matched healthy controls (mean age at colon biopsy, 71.8 ± 6.1 years; 65% were men) were included in this study (Table 1). Patients with PD had lower cognitive function compared to controls (MMSE scores: 26.8 ± 2.1 vs. 29.2 ± 1.8, respectively, *P* < 0.01).

**TABLE 1:** Clinical characteristics of all participants in the current study.

Characteristics	Controls (*n* = 40)	PD (*n* = 51)	*P-*value
Current age (years)	73.7 ± 3.6	76.8 ± 11.1	0.47
Age at biopsy (years)^#^	71.8 ± 6.1	69.2 ± 9.2	0.12
Gender (Male,%)	26 (65.0)	28 (54.9)	0.33
MMSE	29.2 ± 1.8	26.8 ± 2.1	<0.01**
Disease duration (years)	N.A.	6.4 ± 1.9	
Hoehn-Yahr stages (on)	N.A.	2.2 ± 1.4	
Hoehn-Yahr stages (off)	N.A.	3.2 ± 1.3	
UPDRS part III scores (on)	N.A.	15.2 ± 8.7	
UPDRS part III scores (off)	N.A.	27.3 ± 12.6	

Values represent the mean ± standard deviation. PD, Parkinson’s disease; MMSE, mini-mental status examination; N.A., not available; UPDRS, unified Parkinson’s disease rating scale. **P < 0.01.

*^#^The age at the most recent biopsy, for patients that received serial biopsies.*

**FIGURE 2:**
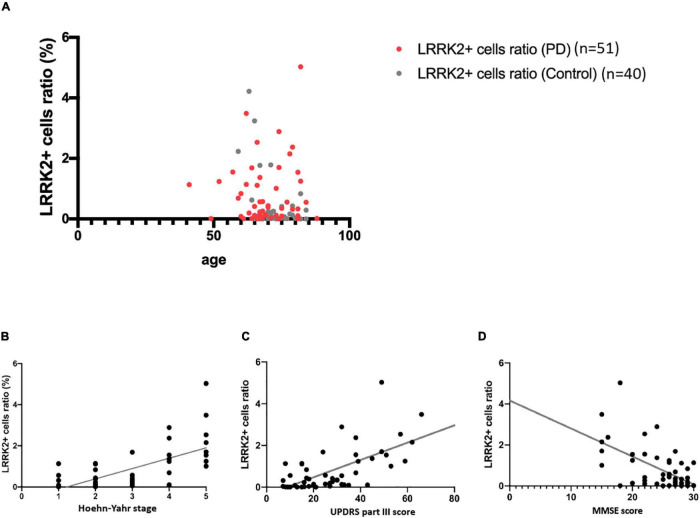
Correlation of colonic LRRK2 expression with clinical severity in PD. **(A)** Age did not correlate with LRRK2 expression in colonic biopsies. **(B,C)** Correlation of colonic LRRK2 expression with Hoehn-Yaht stage and UPDRS part III motor score. **(D)** Correlation of colonic LRRK2 expression with MMSE score.

The fraction of LRRK2-positive cells within the total cell count in colonic biopsies, termed the LRRK2 (+) cell ratio, were not normally distributed in PD patients or controls (Figure 1C). Age did not affect the colonic expression of LRRK2 in these two groups (control group: *r* = –0.1810, 95% confidence interval: –0.4736 to 0.1477, *P* = 0.26; PD group: *r* = 0.0081, 95% confidence interval: –0.2758 to 0.2907, *P* = 0.96; Figure 2A). We observed that LRRK2 immunostaining in the colonic submucosa and lamina propria was significantly higher in patients with PD compared to controls [Figures 1D,E, controls vs. PD patients: 0.45 ± 0.39% (range 0.00–4.22%) vs. 0.81 ± 0.53% (range 0.01–5.03%); *P* = 0.02]. We then genotyped for three common pathogenic mutations (p.R1441C/G/H, p.I2012T, and p.G2019S) and the Asian-specific risk polymorphism (p.G2385R) in *LRRK2*. Among 51 PD patients, three patients carried the *LRRK2* mutations (one with *LRRK2* p.R1441H mutation and the remaining two with *LRRK2* p.I2012T mutations) and 9 had the PD risk p.G2385R polymorphism. Colonic LRRK2 immunoreactivity was higher in those with *LRRK2* genetic variants, especially those with pathogenic mutations, compared to those with wild type *LRRK2* (Figure 1F, *LRRK2* mutation carriers vs. *LRRK2* p.G2385R carriers: 3.49 ± 1.79% vs. 1.73 ± 0.52%, *P* = 0.02; *LRRK2* p.G2385R carriers vs. wild type *LRRK2* carriers: 1.73 ± 0.52% vs. 0.21 ± 0.13%, *P* = 0.01; *LRRK2* risk or mutation carriers vs. wild type *LRRK2* carriers: 2.44 ± 1.15% vs. 0.21 ± 0.13%, *P* < 0.01). Among 40 control participants, 2 controls had *LRRK2* p.G2385R risk variant and none had *LRRK2* pathogenic mutations. The colonic expression levels of these two control participants with *LRRK2* p.G2385R variant were 4.22 and 3.24% individually, which expression levels were higher than the average level of the control participants of 0.45 ± 0.39%. Both controls were in the early fifties and had no signs of parkinsonism feature.

We then further examined the relationship between the LRRK2 (+) cell ratio in colon biopsies and the clinical severity of PD in biopsied samples from 51 patients with PD. For motor symptom severity, the colonic LRRK2 (+) cell ratio correlated with both Hoehn-Yahr stage in the “off” state (*r* = 0.6864, *P* < 0.001) and UPDRS part III motor scores in the “off” stage (*r* = 0.6335, *P* < 0.001; Figures 2B,C). The colonic LRRK2 expression level negatively correlated with the MMSE score (*r* = –0.5774, *P* < 0.001; Figure 2D), suggesting that a higher LRRK2 expression level is associated with worse cognitive function in patients with PD.

**FIGURE 3:**
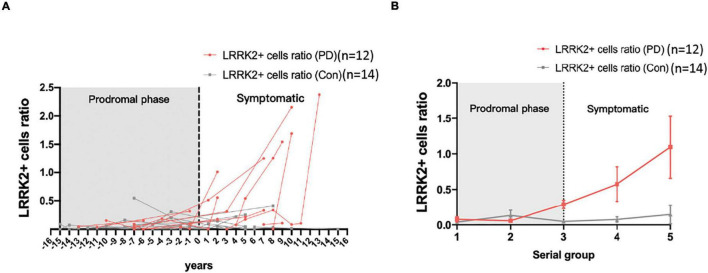
Time-sequential serial analysis of LRRK2 expression in colonic biopsies from patients with PD and controls. **(A)** Individual data for colonic LRRK2 expression from the serial study. **(B)** Grouping analysis of colonic LRRK2 expression was significantly different between PD patients and controls in individual time serial groups.

To further elucidate the time-course changes in colonic LRRK2 expression in the disease process of PD, the colonic tissues biopsied from 12 PD patients who had a series of colon biopsied samples (age at the latest biopsy: 75.1 ± 5.8 years; 58% were men) and 14 age- and gender-matched control participants (age at the latest biopsy: 71.4 ± 4.5 years; 57% were men) who underwent a series of colon biopsies were analyzed. Compared to controls, the LRRK2 expression levels started to increase in patients with PD in the prodromal phase, sampling time for which was before the onset of PD motor symptoms (Figures 3A,B). Looking at the individual data, the tendency for increased LRRK2 expression varied, as some patients had increased LRRK2 levels before the onset of motor dysfunction and some had steady expression regardless of the disease process (Figure 3A). Therefore, the results were further classified into five serial groups according to the sampling time in relation to the age of onset of motor dysfunction in PD group, which was 67 years old. Consistently, the grouping data showed that the colonic LRRK2 expression started to increase before the diagnosis of PD and was higher in PD patients compared to controls in serial group 3 (*P* < 0.01). As PD progressed, the expression of LRRK2 in colonic biopsies increased in accordance with the disease course (Figure 3B).

## Discussion

This study demonstrated an increase in LRRK2 expression in colonic biopsied specimens from patients with PD compared to age- and gender-matched controls. Among PD patients, the colonic LRRK2 level correlated with disease severity in both motor and cognitive function, and the expression levels were higher in those carrying *LRRK2* risk or pathogenic variants than those with wild type *LRRK2*. Furthermore, PD patients in the prodromal phase had increased colonic LRRK2 expression compared to controls and, as the disease progressed, the expression of LRRK2 in colonic tissues progressively increased. These results suggest that colonic LRRK2 expression may serve as a surrogate gut-oriented marker of PD.

Routine colonic biopsies typically comprise mucosal and submucosal tissues, including submucosal immune cells and enteric nervous system ganglia and neurites (Lebouvier et al., 2010). Consistent with previous results, the LRRK2 expression in biopsied tissues was low (Westerlund et al., 2008). LRRK2 was detected in the submucosa and lamina propria, but not in intestinal epithelial cells, which is in line with our findings. Evidence has shown that LRRK2 is highly expressed in a subset of mannose receptor-positive macrophages, dendritic cells, and intestinal B cells (Gardet et al., 2010). Notably, the expression of LRRK2 would be activated by the pro-inflammatory cytokine IFN-γ, which would be upregulated by bacteria-induced or gut inflammation, and LRRK2 is an activator of the pro-inflammatory NF-κB pathway, resulting in a vicious cycle of gut inflammation (Gardet et al., 2010). Our observations show a higher colonic expression level of LRRK2 in PD patients compared to controls, especially in those with *LRRK2* risk or mutation carriers, and the expression correlated with disease severity, suggesting that LRRK2 may be involved in the pathophysiology of PD in the very beginning of the disease process that is relevant to the host responses to altered gut microbiota observed in PD (Lin et al., 2019; Romano et al., 2021). Furthermore, reproducibility of the immunostaining and the storage duration of the biopsied samples are critical issues for this kind of retrospective pathology analysis. As the colon biopsied samples analyzed in this study were collected across the time period of 10 years, the stability of the antigen or the targeted protein degradation remains an issue for immunostaining analysis. Antigen decay in archival formalin-fixed paraffin-embedded (FFPE) tissue sections for immunohistochemistry is a well-known phenomenon which may affect the results of the analysis and the length of storage time appears fundamental. A recent study has shown that the immunoreactivities for cytosolic proteins could be preserved for up to 50 years, but nuclear antigens, for example Ki67 and CD31, presented reduced staining intensity in older and aged FFPE blocks (Grillo et al., 2017). As LRRK2 is a cytosolic protein, we assume the immunoreactivity for those biopsied blocks acquired 10 years ago may not decline dramatically. However, a future prospective follow up study analyzing the immunoreactivity of multiple targeted proteins in fresh biopsied samples is needed to confirm our findings.

Emerging evidence has suggested that PD comprises two subtypes. One subtype is the gut-initiated body-first subtype, in which the Lewy body pathology originates in the gut enteric nervous system and then ascends through the vagus nerve and sympathetic connectome to the brainstem. The second subtype is a brain-first subtype in which the Lewy body pathology initially arises in the brain itself or enters through the olfactory bulb, subsequently descending to the peripheral autonomic nervous system (Horsager et al., 2020). Genetic evidence has suggested that LRRK2 is a link between IBD and PD (Umeno et al., 2011; Zhu et al., 2019), and mild intestinal inflammation is common in patients with PD (Forsyth et al., 2011); therefore, it is reasonable to speculate that the colonic LRRK2 expression would be increased in those who are in the body-first subtype of PD. The serial analysis of colonic biopsies in this study showed a diverse pattern of colonic LRRK2 expression from the prodromal phase to the symptomatic stage years after the occurrence of motor dysfunction. Some patients had increased LRRK2 expression compared to healthy controls before the diagnosis of PD, and the LRRK2 level increased along with the disease progression, whereas a minority of the patients did not follow this trend and possessed steady, low LRRK2 levels throughout the disease course. Although Braak’s pathology staging showed that Lewy body pathology initially forms in the gut enteric nervous system (Braak et al., 2003a), alternative neuropathology staging systems for Lewy pathology in some PD patients has shown an amygdala-predominant profile with significantly fewer Lewy bodies in the brainstem or gut, which fits in the brain-first subtype of PD (Raunio et al., 2019). Therefore, we speculate that those with abundant colonic LRRK2 expression or an increased colonic LRRK2 level in the prodromal phase may have associated Lewy body pathology in the gastrointestinal tract. Of note, there were two control participants carrying the *LRRK2* p.G2385R risk variant and having the relatively high levels of colonic LRRK2 expression. Although these two controls did not show evidence of parkinsonism features in their early fifties while receiving colonic biopsy, they should be closely monitored for early prodromal or motor symptoms of PD during follow-up.

Further studies that examine the co-immunostaining of a-synuclein and LRRK2 in those with preceding gastrointestinal symptoms, brainstem dysfunctional symptoms of rapid eye movement sleep behavior disorder or having *LRRK2* risk variants are needed to confirm our hypothesis.

This study had several limitations. First, although we have a large number of colonic biopsied samples for cross-sectional comparison study but only a relatively small number of patients had a series of colonic biopsies in our serial change study. In addition, the participants did not usually undergo serial colonic biopsies at regular intervals, which made time grouping necessary to avoid a lack of data in a certain year influencing the entire analysis. Second, the biopsied samples were taken during routine random colon biopsy and the biopsied anatomic sites may affect the results. Third, in this retrospective study, we did not have the gut symptoms or gastrointestinal questionnaire data of all enrolled participants. Future prospective studies with comprehensively gastrointestinal symptoms combing with food questionnaire are needed. Finally, we did not examine the colonic expression of LRRK2 in different cell types or examine any evidence of gut inflammation. Further studies examining the LRRK2 expression in different neuronal subtypes in the colon, such as choline acetyltransferase (ChAT)-positive, vasoactive intestinal peptide (VIP)-secreting neurons and sympathetic tyrosine hydroxylase-positive neurons, will provide a more comprehensive landscape of LRRK2 expression in individual cell types of PD process. Future studies that investigate the expression of LRRK2 and a-synuclein in individual colonic cell types and inflammatory marker are needed.

## Conclusion

In conclusion, this study showed that LRRK2 expression is increased in colonic biopsies from PD patients, and the expression level was associated with the clinical severity of PD. Future prospective studies with a larger sample size of participants are needed to confirm our findings.

## Data Availability Statement

The original contributions presented in the study are included in the article, further inquiries can be directed to the corresponding author/s.

## Ethics Statement

This study protocol was approved by the Institutional Board Committee of National Taiwan University Hospital. The patients/participants provided their written informed consent to participate in this study.

## Author Contributions

P-HL, H-LC, and C-HL: study concept and design. P-HL, H-LC, C-TS, H-MC, M-SW, and C-HL: acquisition of data. P-HL, H-LC, J-FH, and C-HL: analysis and interpretation of data. P-HL and C-HL: drafting the manuscript. C-HL: critical revision of the manuscript for important intellectual content, and study supervision. P-HL: statistical analysis. All authors contributed to the article and approved the submitted version.

## Conflict of Interest

The authors declare that the research was conducted in the absence of any commercial or financial relationships that could be construed as a potential conflict of interest.

## Publisher’s Note

All claims expressed in this article are solely those of the authors and do not necessarily represent those of their affiliated organizations, or those of the publisher, the editors and the reviewers. Any product that may be evaluated in this article, or claim that may be made by its manufacturer, is not guaranteed or endorsed by the publisher.
